# Ultrasound-Assisted Optimization of the Activation and Inactivation of Thermostable α-Amylase

**DOI:** 10.3390/ijms27083503

**Published:** 2026-04-14

**Authors:** Zahra Azzouz, Ourdia-Nouara Kernou, Naima Djerroud-Mohellebi, Festus Ogungbemiro, Zahir Amghar, Nassima Kichi, Azzeddine Bettache, Nawel Boucherba, Samir Hadjal, Patricia Rijo

**Affiliations:** 1 Laboratoire de Microbiologie Appliquée (LMA), Faculté des Sciences de la Nature et de la Vie, Université de Bejaia, Bejaia 06000, Algeria; zahir.amghar@univ-bejaia.dz (Z.A.); nassima.kichi@univ-bejaia.dz (N.K.); azzeddine.bettache@univ-bejaia.dz (A.B.); nawal.boucherba@univ-bejaia.dz (N.B.); 2 Laboratoire de Biomathématiques, Biophysique, Biochimie et Scientométrie (L3BS), Faculté des Sciences de la Nature et de la Vie, Université de Bejaia, Bejaia 06000, Algeria; ourdia.kernou@univ-bejaia.dz; 3 Département Ecologie et Environnement, Faculté des Sciences Biologiques, Université des Sciences et de la Technologie Houari Boumediene USTHB Bab-Ezzouar, Alger 16111, Algeria; naima.djerroud@univ-bejaia.dz; 4 CBIOS—Centro de Investigação em Biociências e Tecnologias da Saúde, Universidade Lusófona, 1749-024 Lisboa, Portugal; f8269@ulusofona.pt; 5 Centre de Recherche en Technologie Agroalimentaire, Route de Targua-Ouzemour, Bejaia 06000, Algeria; 6 Laboratoire de Recherche et Développement Produit, Direction de Recherche et Développement, Cevital Agro-Industrie, Nouveau Quai Port de Bejaia, Bejaia 06000, Algeria; samir.hadjal@cevital.com; 7 Centro de Química Estrutural, Instituto de Ciências Moleculares, Universidade de Lisboa, 1749-016 Lisboa, Portugal; 8 iMed.ULisboa—Instituto de Investigação do Medicamento, Faculdade de Farmácia, Universidade de Lisboa, 1649-003 Lisboa, Portugal

**Keywords:** ultrasound, thermostable α-amylase, optimization, activation, inactivation

## Abstract

Ultrasound is a non-thermal technology increasingly applied in food processing to modulate enzyme activity. This study investigated the effects of ultrasonic irradiation on the activity of a commercial thermostable α-amylase. Enzyme activity was determined by quantifying reducing sugars released from starch using the 3,5-dinitrosalicylic acid method, and protein concentration was measured by the Bradford assay. A one-factor-at-a-time approach was used to evaluate the effects of ultrasonic amplitude, treatment time, enzyme concentration, incubation temperature, and calcium ion concentration. Subsequently, a Box–Behnken design was applied to analyze the combined influence of amplitude, treatment duration, temperature, and calcium concentration on residual activity. The enzyme exhibited an initial activity of 46.27 ± 3.63 U/mL under standard assay conditions. Moderate ultrasonic amplitudes enhanced activity, whereas prolonged exposure and elevated temperatures promoted inactivation. Statistical analysis showed that the incubation temperature and calcium concentration significantly influenced residual activity, and the quadratic model provided a good fit (R^2^ = 0.94). Optimal inactivation conditions were identified at 60% amplitude, 9 min treatment, 85 °C, and 9 ppm calcium, resulting in 66.3% enzyme inactivation. These findings support the use of ultrasound-assisted processing as a controllable strategy to regulate thermostable α-amylase activity in industrial enzyme applications.

## 1. Introduction

Ultrasound has emerged as an important non-thermal technology for process intensification in food and biotechnological applications. Unlike conventional thermal treatments, ultrasonic processing relies on acoustic cavitation, which can generate localized high-energy microenvironments and mechanical effects (e.g., microstreaming and shear forces) capable of influencing reaction kinetics and biomolecular systems without uniformly increasing bulk temperature [1,2,3]. This combination of mechanical and physicochemical effects has motivated increasing interest in ultrasound-assisted processing as a strategy to improve efficiency and reduce processing severity.

In food processing, ultrasound has been applied to operations such as extraction, preservation, cleaning, degassing, cutting, and the inactivation of microorganisms and enzymes [4,5,6,7,8]. In industrial biotechnology, it has also been used to enhance enzyme-assisted extraction and improve the recovery and bioactivity of plant-derived compounds [9,10]. Depending on operating conditions (including amplitude, treatment time, temperature, and medium composition), ultrasound may either enhance enzymatic performance—often associated with improved mass transfer and changes in enzyme–substrate interactions—or promote enzyme inactivation through cavitation-related stresses, localized heating (“hot spots”), and sonolysis-derived reactive species [11]. For this reason, establishing robust operating windows is essential when ultrasound is applied to enzymatic systems.

Thermostable α-amylase, commonly produced by Bacillus licheniformis, is widely used in the food and fermentation industries due to its capacity to catalyze starch hydrolysis into fermentable sugars. Industrial starch conversion typically involves two sequential enzymatic stages: liquefaction, catalyzed by α-amylase, and saccharification, mediated by glucoamylase [12]. Increasing α-amylase efficiency is desirable for processes such as brewing, baking, and bioethanol production, where improved hydrolysis performance can enhance productivity and process economics [13]. However, the high thermostability of α-amylase, while advantageous during high-temperature liquefaction, can also be problematic. Residual thermostable α-amylase activity may persist after conventional thermal processing and can adversely affect product quality and stability in downstream steps, making controlled modulation and/or inactivation industrially relevant [13].

Optimization approaches based on varying one factor at a time are limited because they do not capture interactions between process variables. In contrast, response surface methodology provides a multivariate statistical framework that can model combined effects and efficiently identify optimal operating conditions for complex processes [14]. Although ultrasound has been broadly explored in food processing, direct and systematically optimized modulation of commercial thermostable α-amylase—considering both activation and inactivation objectives—remains comparatively limited, particularly in an industrially relevant parameter space.

In this study, a commercial thermostable α-amylase was selected as a model system to evaluate the ultrasound-assisted modulation of enzyme activity. The objective was to investigate the effects of ultrasonic parameters on α-amylase activation and inactivation and to determine the optimal conditions using a Box–Behnken experimental design. We hypothesized that α-amylase activity would respond in a parameter-dependent manner, with activity enhancement under moderate ultrasonic conditions and increased inactivation under more severe thermal–acoustic conditions, particularly as a function of incubation temperature and calcium ion concentration.

## 2. Results and Discussion

### 2.1. Thermostable α-Amylase Inactivation Optimization Using the Classical OFAT Method

The protein concentration of the commercial enzyme preparation was determined to be 19.36 mg/mL. Under standard assay conditions (80 °C, pH 7.0, without ultrasound pre-treatment), the initial enzymatic activity was 46.27 ± 3.63 U/mL.

Ultrasound is known to exert dual effects on enzymes, potentially enhancing or reducing catalytic activity depending on processing parameters such as amplitude, exposure time, temperature, and medium composition [3]. These effects are generally associated with acoustic cavitation phenomena, which may influence enzyme–substrate interactions, mass transfer, and structural stability [15,16].

To evaluate the individual contribution of key variables, a classical one-factor-at-a-time (OFAT) approach was employed. The parameters investigated included ultrasound amplitude, treatment time, enzyme concentration, metal ions, and incubation temperature.

#### 2.1.1. Ultrasonic Amplitude Effects on Enzyme Activity

**Figure 1:**
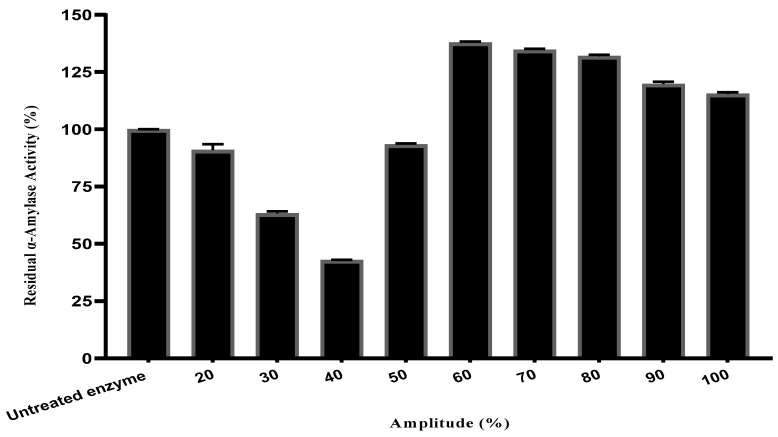
Effect of ultrasound amplitudes on thermostable α-amylase enzyme activity.

The effect of ultrasound amplitude on thermal residual α-amylase activity after a 5 min treatment is shown in Figure 1; the untreated enzyme sample incubated under standard assay conditions (80 °C, pH 7, without ultrasound) was used as the control and its activity was taken as 100% residual activity. The amplitude range tested (20–100%) was determined by the operational specifications of the Vibra Cell VCX sonicator, which has a minimum functional amplitude of 20% and a maximum of 100% of its output capacity. Amplitudes below 20% cannot be reliably generated by this equipment and were therefore not included in the experimental design.

The amplitude of ultrasound treatment plays a critical role in the regulation of enzyme activity, with both activation and inactivation depending on the level of ultrasound treatment applied.

The significant enzyme activity increase was achieved at an amplitude of 60%, 70% and 80%, with 137.8%, 134.6% and 131.9% residual activity, respectively. With these values, the highest increase in α-amylase activity compared to the untreated control (100%) was observed. This activation is probably due to moderate cavitation effects, which induce beneficial conformational changes in the enzyme, enhancing its interaction with the substrate. Conversely, the amplitudes of 20%, 30% and 40% produced decreasing levels of residual activity for 90.9%, 63.3% and 42.8%, respectively, indicating partial inactivation. Residual activity was lowest at 40% amplitude, suggesting that cavitation forces under these conditions may have caused structural damage or conformational changes in the enzyme. However, higher amplitudes (90% and 100%) also preserved high levels of activity (119.8% and 115.5%, respectively), suggesting that above a certain energy cut-off, the enzyme is reactivated by conformational rearrangements.

The observed effects can be explained by ultrasound-induced mechanisms. At adequate intensities and frequencies, the ultrasonic waves create cavitation, magneto-striction effects with mechanical oscillations, enhancing the enzyme–substrate interactions by changing the enzyme conformation temporarily [17]. However, too much ultrasonic energy can deactivate enzymes in two main ways: by strong forces from collapsing cavitation bubbles and by creating free radicals when water is broken down by sound waves [18]. Free radicals and shock waves can denature protein structures, and cavitation bubbles collapse at gas–liquid interfaces in particular [19,20]. Subsequent cycles between compression and rarefaction of ultrasound waves generate microfluxes and turbulence, which cause the disruption of secondary and tertiary structures, reducing enzymatic activity slightly beyond a tolerable threshold [21,22,23].

Maser, Maiyah [24] optimized the ultrasound-assisted extraction of antidiabetic compounds from green leafy vegetables and reported that an amplitude of 80% combined with 30 minutes of sonication yielded the highest enzyme inhibitory activity and extraction efficiency. Their findings support the selection of 80% amplitude as an effective operating condition in ultrasound-based studies.

The ANOVA statistical analysis showed a significant effect of amplitude on α-amylase activity, with a *p*-value < 0.05, which confirms the reliability of these observations.

#### 2.1.2. Activity Effect of Ultrasound Treatment Time on Enzyme Activity

**Figure 2:**
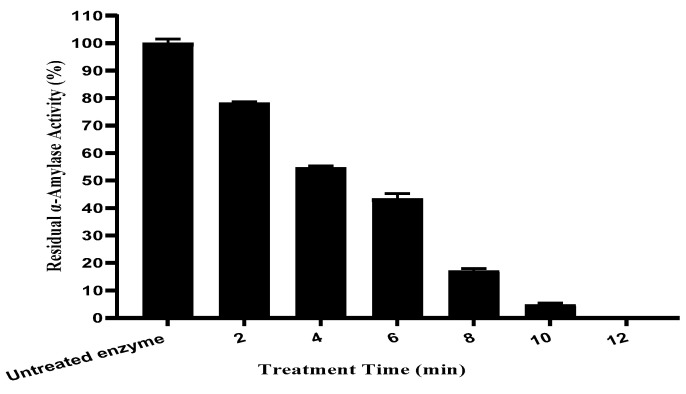
Formatting effect of ultrasound treatment duration on thermostable α-amylase enzyme activity.

The effect of sonication time on α-amylase activity was studied under a constant ultrasonic amplitude of 80%. The amplitude of 80% was selected for this time course experiment because the effect of amplitude showed that the 60–80% range produced the highest activation of α-amylase, thus representing a relevant compromise for studying time-dependent effects. Figure 2 shows that increasing the duration of treatment induced a progressive decline in enzyme activity relative to the control (0 min), resulting in complete inactivation for durations of 10 min or more. However, these findings show that extended exposure to ultrasound has a negative effect on the enzymes’ integrity and functionality.

Moreover, enzyme activity loss over time is attributed to the multiple factors associated with ultrasound treatment, including excessive mechanical shear forces, localized heating, increased pressure and water sonolysis free radical generation [25].

All these factors disturb the enzyme’s native structure and reduce its catalytic efficiency. Ultrasonic techniques for modifying the secondary and tertiary structures of proteins, for increasing hydrophobic surface exposure, and for protein aggregation are all factors that can contribute to reduced activity [16]. The main cause of these effects is the shock waves and microfluxes produced by the shock waves. As a result, the enzyme becomes less water-soluble, and buried hydrophobic residues become exposed, leading to structural instability and activity loss [26,27].

These findings are in agreement with Kapturowska, Stolarzewicz [16], who studied the lipolytic activity of enzymes treated in a horn sonicator. Using cavitation-induced denaturation, scientists reported a complete loss of enzyme function, indicating that high-intensity ultrasonic waves can cause irreversible structural damage and complete inactivation of the enzyme.

The results of this study overall indicate that while ultrasonic treatment is effective in enhancing α-amylase activity under specific conditions, prolonged exposure results in significant inactivation, due probably in part to structural changes and destabilization of the enzyme’s active conformation.

#### 2.1.3. Effect of Enzyme Concentration

**Figure 3:**
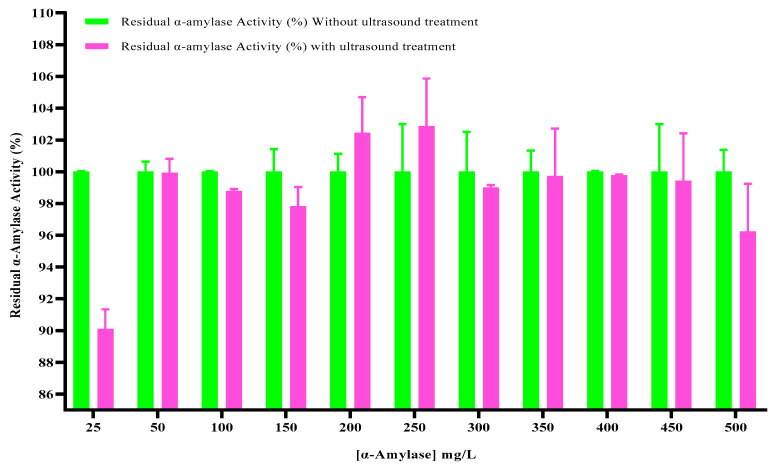
Effects of thermostable α-amylase enzyme concentration under ultrasonic treatment.

The effect of the enzyme concentration on α-amylase activity is shown in Figure 3. Enzyme activity remains the same in the presence and absence of irradiation at concentrations of 50, 100, 300, 350, 400 and 450 mg/L (Figure 3). However, a slight decrease was observed in α-amylase activity at 25, 100, 150, and 500 mg/L and a small increase at 200 and 250 mg/L.

Statistical analysis using one-way ANOVA revealed that variations in enzyme concentration ranging from 25 to 500 mg/L did not significantly affect residual α-amylase activity following ultrasound exposure (*p* > 0.05). Similar activity levels were observed across all concentrations, indicating that the enzyme concentration was not a determining factor in ultrasound-induced activation or inactivation. This suggests that the effects of ultrasound are primarily governed by cavitation-related mechanical forces rather than enzyme availability, indicating that the enzyme concentration did not substantially influence the observed outcomes.

The enzyme concentration has a significant influence on enzyme activity. Overall, increasing the enzyme concentration results in increased enzyme activity up until a saturation level is achieved. Indeed, a larger enzyme concentration results in a higher chance of reaching a substrate, favoring the enzymatic reaction. However, at concentrations over a certain range, the enzyme activity level remains stable or decreases due to the saturation of substrate or inhibition due to high concentrations of reaction products or by-products [28]. In that case, the effects of ultrasound can predominate and lead to a decrease in enzymatic activity [29].

#### 2.1.4. Metal Ion Effect

**Figure 4:**
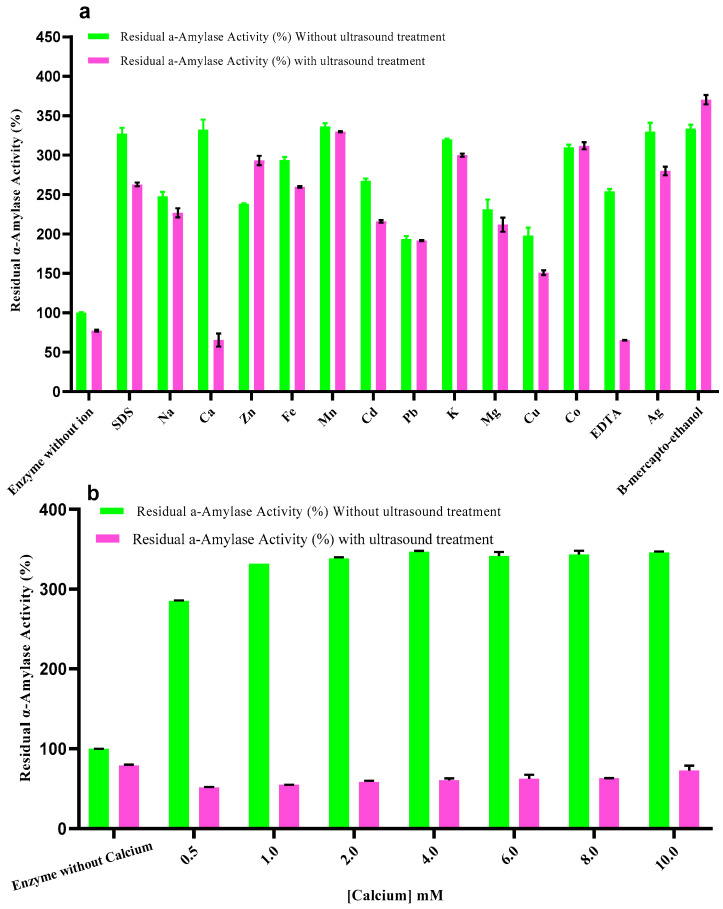
Effects of metal ions, chemical reagents (**a**), and calcium concentration (**b**) on the activity of thermostable α-amylase with and without ultrasound treatment.

The impact of different metal ions and chemical reagents on the activity of α-amylase before and after ultrasound treatment is shown in Figure 4a. Whatever the conditions tested, a significant overall increase in enzyme activity was observed compared with the ion-free control in both the untreated and ultrasound-treated enzyme preparations. A complex interplay between metal ions, chemical additives and ultrasonic treatment in modulating the catalytic function of α-amylase is suggested.

Zn^2+^ appears likely to play a structural role in stabilizing the active conformation of the enzyme [30], and β-mercaptoethanol reduces disulfide bonds, thus potentially enhancing enzyme flexibility and reactivity under ultrasonic conditions [31].

In contrast, a significant decrease in enzyme activity was observed with Ca^2+^ and EDTA under ultrasound exposure, corroborating the findings from prior research [26,27,28]. Ca^2+^, although generally associated with α-amylase stabilization, appears to have a denaturing or destabilizing effect under ultrasound, reducing residual enzyme activity significantly (by nearly 74% in the presence of Ag^+^ and 25.7% with EDTA).

The results, however, confirm that α-amylase is a metalloproteinase, where the presence of metal ions is a critical factor in the stability and activity of the enzyme [32].

EDTA, a chelating agent that has been widely used, shows strong inhibitory effects at a concentration of 1 mM, when sonication is applied by contrast, via means of critical metal ions such as Zn^2+^ and Ca^2+^ to be sequestered selectively. The effect of this disruption in the binding sites of the metal ions is likely to modify enzyme tertiary structure and inhibit its catalysis activity [33].

In the same way, SDS anionic detergent inhibits the activity of α-amylase significantly under ultrasonic; SDS is known for protein denaturing, hydrophobic interaction interference and electrostatic environment modification [33,34,35].

Heavy metals including Cu^2+^, Ag^+^ and Cd^2+^ are reported to inhibit the α-amylase activity observed in this study and previously reported by Prieto, Bort [36], Gupta, Gigras [37] and Mihoub, Chaoui [38]. Taken together, these ions are able to bind to the enzyme, to induce changes in conformation in the enzyme and/or to block the catalytic site. However, only moderate inhibition is caused by Mn^2+^, Co^2+^, and Pb^2+^, indicating a less disruptive interaction with the enzyme structure.

Figure 4b has a closer exploration into the relation between the calcium concentration and residual α-amylase activity following sonication treatment using ultrasound. In addition, in the absence of substrate, raising the Ca^2+^ concentration from 0 to 10 mM resulted in a general reduction in residual activity under sonication compared with unsonicated controls. For instance, at 0 mM Ca^2+^, activity drops from 100% (control) to 79% with ultrasound, and this reduction is more pronounced at higher calcium levels, reaching a minimum of 51.4% at 0.5 mM and 55% at 1 mM Ca^2+^. Without ultrasound, activity appears artificially elevated due to the absence of substrate, as seen in the anomalously high residual activity values (332–346%).

These observations support the notion that ultrasound can destabilize enzyme–metal interactions, especially in metalloproteins like α-amylase. While calcium is essential for stabilizing the enzyme under normal conditions, sonication appears to compromise this protective effect. The enzyme may be disrupted by cavitation-induced shear forces or local heating, which disrupts calcium binding or alters the conformation of the enzyme [32,39].

#### 2.1.5. Effect of Incubation Temperature

**Figure 5:**
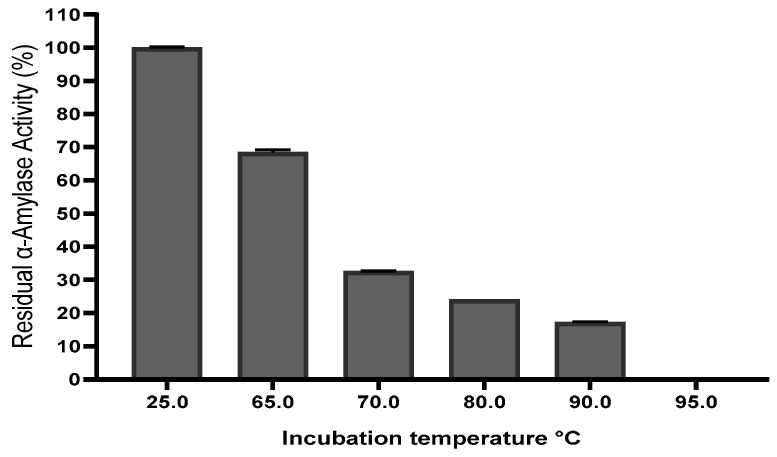
Effect of incubation temperature on α-amylase activity with ultrasound treatment.

The incubation temperature of the enzyme prior to ultrasound treatment was evaluated over a temperature gradient to determine its influence on the catalytic performance of thermostable α-amylase (Figure 5). The activity of the enzyme at 65 °C decreased moderately to around 68% of the residual activity. Temperatures of 70 °C, 80 °C and 90 °C gave rise to a significant reduction in enzyme activity, corresponding to 30%, 22% and 18% respectively. A temperature of 95 °C showed a complete absence of enzymatic activity, corresponding to thermal denaturation that is ongoing at this temperature. This indicates that while the enzyme maintains stability up to 65 °C, both its structural integrity and catalytic efficiency are severely impaired at higher temperatures.

Ultrasonic cavitation is effective at high temperatures due to the increased pressure of water in cavitation bubbles, which can induce the denaturation of α-amylase by overheating [17]. High temperatures also increase the susceptibility of the enzyme to thermal degradation, as the pressure of vapor afflicts the cavitation of ultrasonical bubbles [20]. High temperatures promote the filling of cavities by solvent vapor, reducing their adiabatic enforcement force. Conversely, lower temperatures increase the viscosity of the environment, limiting the propagation of acoustic ones [33]. Ultrasound cavitation causes water sonolysis, generating hydroxyl radicals and hydrogen atoms that disrupt the α-amylase enzyme structures, impacting their catalytic activity and potentially leading to their inactivation [33,34]. Ultrasound only induces a significant decrease in enzyme activity without completely inactivating them. However, when combined with high-temperature thermal treatment, they become more effective in inactivating the enzyme, exploiting the synergies between ultrasounds, heat, and pressure for optimal enzymatic inactivation [23,35].

### 2.2. Box–Behnken Analysis of Thermostable α-Amylase Inactivation-Based RSM Design

To refine the optimization and evaluate the interaction effects, a Box–Behnken design was applied using four key variables identified from the OFAT experiments: amplitude (A), treatment duration (B), incubation temperature (C), and calcium concentration (D). Table 1 displays the Box–Behnken design (BBD) matrix and related RSM experiment results. This study evaluated the effects of ultrasound on α-amylase activity using four key variables, A, B, C and D. The initial values of these parameters were based on the optimal levels found in early OFAT trials.

#### 2.2.1. Model Performance and Fitting Using RSM

The BBD then refined these parameters and looked at how they affected enzyme activity on their own and together. Table 2 shows the analysis of variance (ANOVA) for the quadratic model fitted to the residual α-amylase activity (%) obtained in the BBD experiments (Table 1). The statistical analysis was conducted using Design-Expert software (Version 13.0.5.0 Stat-Ease Inc.) with the reported F- and *p*-values derived from the ANOVA of the BBD model based on the complete set of experimental responses shown in Table 1. The model was very important because it had a model F-value of 15.15 and a *p*-value less than 0.0001 (Table 2). This means that there is only a 0.01% chance that the outcome was due to noise. This shows how well the model predicts how changes in ultrasonography parameters will affect α-amylase activity.

The factors tested with statistically significant effects (*p* < 0.05) were the quadratic term C^2^ (incubation temperature vs incubation temperature), calcium concentration (D) and incubation temperature (C), indicating their critical role in α-amylase activity. In the BC interaction (treatment time × incubation temperature), the effect was also significant (*p*-value = 0.0272), showing a combined effect of these two parameters on enzymatic activity. Other terms, such as amplitude (A), treatment time (B), A^2^ (amplitude^2^), B^2^ (treatment time^2^) and D^2^ (calcium concentration) and most of the interactions (AB, AC, AD, BD, CD), were statistically insignificant (*p* > 0.05) (Table 2), suggesting a smaller contribution to the overall response of the model under the conditions tested.

The F-value of 0.7065 and *p*-value of 0.7124 for the lack-of-fit test confirm that the model’s lack of fit is not significant with respect to the pure error. This implies that the model is well-fitted and that random error, not any systematic deviation, is primarily responsible for the observed variation. The results obtained are in accordance with earlier research showing that a significant model with non-significant lack of fit is ideal for a credible response surface analysis [40].

The model was able to account for 94.65% of the variation in α-amylase activity, with a coefficient of determination (R^2^) of 0.9465 (Table 2). The model is adequate in explaining most of the variability in the trial results since the R^2^ value is greater than 0.75 [41]. The adjusted R^2^ (0.8840) and predicted R^2^ (0.7331) (Table 2) further support the model’s predictive accuracy and robustness.

**Figure 6:**
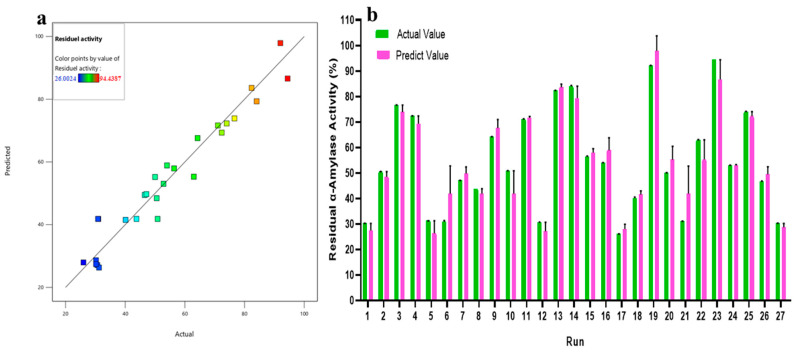
Correlation between predicted and actual values of α-amylase activity (**a**), and their distribution (**b**) based on the Box–Behnken design (BBD).

Figure 6 indicates a strong relationship between the actual and anticipated values of α-amylase activity. The data points are close together along the diagonal line. The accuracy of the model and its ability to predict results under different ultrasonic processing circumstances may be seen in the linear plot of the data. Additionally, the R^2^ value of 87.38% indicates a strong correlation between the model and the data. This paradigm may be used to navigate the design space. Therefore, considering the statistical features, it can be inferred that the model is sufficient for determining the main effects of the components [42].

The final model of the second-order polynomial quadratic regression equation (Equation (1)), expressed in terms of coded factors, is given as follows:

Residual activity (U/mL) = 24.00 + 0.83A − 1.43B − 11.92C + 22.68D 
+ 0.25AB + 2.00AC + 0.75AD + 8.25BC − 3.71BD − 3.50CD + 3.51A2 
+ 4.61B2 + 12.38C2 + 2.99
(1)

where A: amplitude, B: treatment time, C: temperature, and D: calcium concentration. A positive sign in front of the terms indicates a synergistic effect, while a negative sign indicates an antagonistic effect. Negative values of an estimated coefficient indicate a negative influence of the parameters on enzymatic activity.

#### 2.2.2. RSM Analysis of the Interactions Between Influencing Factors

**Figure 7:**
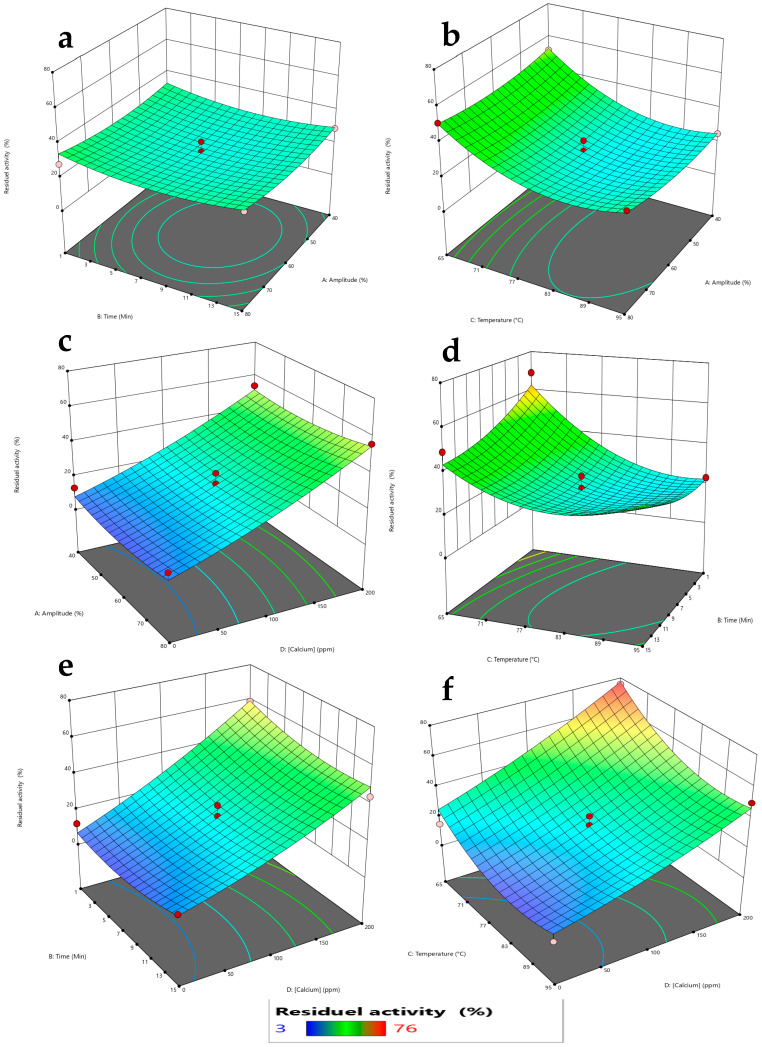
Response surface plots showing interaction effects on ultrasound-modulated α-amylase activity illustrating both activation and inactivation responses. (**a**): Interaction between amplitude (A) and treatment duration (B), (**b**): interaction between amplitude (A) and incubation temperature (C), (**c**): interaction between amplitude (A) and calcium concentration (D), (**d**): interaction between treatment duration (B) and incubation temperature (C), (**e**): interaction between treatment duration (B) and calcium concentration (D), (**f**): interaction between incubation temperature (C) and calcium concentration (D).

To investigate the effects of the different factors and their interactions on the activity of thermostable α-amylase under ultrasound, three-dimensional response surface plots were made (Figure 7a–f). These plots provide visual representations of how two variables interact while keeping the others constant at their central levels. According to the ANOVA results (Table 2), the most significant factors influencing residual α-amylase activity were the incubation temperature (C) and calcium concentration (D), with *p*-values < 0.0001. In contrast, amplitude (A) and treatment time (B) did not show significant individual effects (*p* > 0.05). Among the interaction terms, only BC (time × temperature) was statistically significant (*p* = 0.0272), while all other interactions, AB (*p*-value = 0.9405), AC (*p*-value = 0.5535), AD (*p*-value = 0.8230), BD (*p*-value = 0.2796) and CD (*p* = 0.3071), were not significant.

In Figure 7a, the residual activity of α-amylase after ultrasound treatment is presented as a function of amplitude (A) and treatment duration (B). The plot reveals a slight overall stability in enzyme activity across the tested range of these parameters. This lack of substantial variation corroborates the statistical finding that neither amplitude nor treatment time, nor their interaction, exerted a significant effect on residual activity. In other words, the stability observed in the response surface confirms that both factors and their interaction are non-significant, with ultrasound altering the secondary and tertiary structures of enzymes [22]. Ultrasound affects the enzyme by disrupting hydrogen and Van der Waals bonds, increasing the activation energy at low frequencies [26,43]. For the interaction between amplitude (A) and temperature (C) (Figure 7b), increased amplitude increases residual activity, while higher temperatures decrease it, due to reduced adiabatic collapse strength and thermal denaturation effects [44,45]. At each amplitude level (A), a high calcium concentration (D) (Figure 7c) increases residual activity. Regarding the interaction between time (B) and temperature (C) (Figure 7d), residual activity increases at lower temperature ranges (65–70 °C) across all tested durations (1–15 minutes). In contrast, as the temperature rises (75–95 °C), residual activity decreases regardless of the time interval, suggesting that the enzyme maintains structural stability under mild thermal conditions and can benefit from ultrasound-induced enhancement in mass transfer and substrate accessibility. Prolonged exposure to ultrasound significantly reduces enzyme activity [46,47]. The interaction between time (B) and calcium concentration (D) (Figure 7e) shows that residual activity is maximal at 200 ppm calcium and across all tested durations (1–15 minutes), decreasing with the decreasing calcium concentration. For the interaction between the temperature (C) and calcium concentration (D) (Figure 7f), the results show that residual activity decreases with the increasing temperature when calcium concentrations are low. This suggests that under thermal stress, the enzyme is more susceptible to denaturation in the absence of sufficient calcium. But when the temperature drops and the calcium level rises, residual activity goes up, which suggests that calcium helps keep the enzyme stable at lower temperatures. As temperatures rise, this protective effect becomes less obvious and residual activity decreases regardless of the amount of calcium present. The effect of calcium works better at lower temperatures, but is temperature-dependent.

#### 2.2.3. Validation of the Model

To confirm the predictive performance and reliability of the quadratic model developed through response surface methodology (RSM), a validation was conducted using six experimental trials for α-amylase activation and two for inactivation, as presented in Table 3. These trials were performed under various combinations of ultrasound parameters: amplitude, incubation time, temperature, and calcium concentration.

In the activation phase, all six conditions caused a significant increase in α-amylase activity, with residual activities ranging from 153% to 229%. The greatest activation (229%) occurred when the ultrasonic settings were moderate: 40% amplitude, 5 min incubation, 30 °C and 110 mM calcium (solution 2). Increased temperatures make this effect less significant, and residual activity decreases regardless of the amount of calcium present. The effect of calcium works better at lower temperatures but is temperature-dependent. These studies demonstrate that ultrasound and calcium ions synergistically enhance enzyme activity, particularly at mild to moderate temperatures and low to mid-range amplitudes.

For the inactivation trials, the two experimental conditions led to complete inactivation of the enzyme, with residual activity reduced to 1%. These results were obtained under extreme thermal and ionic conditions: incubation temperature of 90 °C, absence of added calcium (0 mM) and short sonication time (2 to 3 minutes) at an amplitude of 50 to 60%.

The combination of high temperature and the absence of calcium probably contributed to the enzyme’s structural destabilization, which confirms the model’s ability to predict the deactivation of the enzyme when severe ultrasonic parameters are applied.

The results of this validation confirm that the quadratic model effectively records the two-fold behavior of α-amylase under ultrasonic treatment. The model accurately predicts the activation of the enzyme under moderate and calcium-rich conditions, and its inactivation at elevated temperatures and in calcium-free environments. This model is a reliable and versatile tool for adapting ultrasound-assisted processes to specific enzyme performance aims, both to enhance or inhibit activity.

## 3. Materials and Methods

### 3.1. Determination of α-Amylase Activity and Protein Assay

The activity of α-amylase was measured using a modified version of the [48] method [11], a method that quantifies reducing sugars. Their reaction mixture was composed of 900 μL of a 2% starch (Sigma-Aldrich, St. Louis, MO, USA) solution prepared in 50 mM phosphate buffer (pH 7), and an additional 100 μL of enzyme solution. The mixture was incubated in a water bath (Cole-Parmer, WBS-300-120 Shaking Water Bath; 120 VAC, 60H, Vernon Hills, IL, USA) at 80 °C for 10 minutes. Then, 1.5 mL of DNS (3,5-Dinitrosalicylic acid 98%, Sigma-Aldrich, St. Louis, MO, USA) reagent was added, by heating the solution to 100 °C for 5 minutes and then rapidly cooling it in ice-cold water for 5 minutes. Next, the sample was centrifuged (Rotina 380R, Tuttlingen, Germany) at 13,000 rpm for 1 minute to filter out any residual starch particles. Supernatant absorbance was measured at 540 nm using a UV—Visible spectrophotometer (Shimadzu UV mini 1240, Kyoto, Japan). Each measurement was carried out in triplicate. Residual enzyme activity (%) was calculated by comparing the activity of the enzyme after ultrasound treatment with that of the untreated control (taken as 100% activity), using the following formula:

**Residual activity (%)** = (α-amylase Activity after treatment)/α-amylase
Activity of untreated enzyme) × 100
(2)

With this approach, the effect of ultrasound treatment on the stability of the enzyme’s function can be quantified. The activity measurements were carried out in triplicate.

The concentration of protein in the enzyme was assessed using the [49] assay. The standard used was bovine serum albumin (BSA) and the Bio-Rad reagent was used to develop the color. The colorimetric method was based on the binding of Coomassie Brilliant Blue G-250 to aromatic and hydrophobic protein residues, resulting in a measurable shift in absorbance at 595 nm [49].

### 3.2. Thermostable α-Amylase Activity Optimization Using the Classical One-Factor-at-a-Time Approach

The activation and inactivation of thermostable α-amylase was initially optimized using the classic “one-factor-at-a-time” (OFAT) approach, in which one parameter is modified while all others are held constant at predefined levels [42].

To study the effect of ultrasonic amplitude, ultrasonic treatment of a solution of 26 mg/mL enzyme in phosphate buffer (50 mM, pH 7) was carried out using a sonicator (Vibra Cell VC334, Newtown, CT, USA).

The ultrasound amplitude was considered as an experimental factor and was defined according to the operating parameters of the ultrasonic device. The sonicator expresses and controls amplitude exclusively as a percentage (%) of its maximum operating capacity. Amplitudes ranged from 20% to 100%, with pulse cycles of 5 s on and 2 s off, applied over a total duration of 4 minutes, the enzymatic activity of which was assessed by measuring the concentration of reducing sugars.

The time taken for sonication is a vital parameter influencing α-amylase activity. The sonication time on enzymatic activity was tested at different irradiation times (0, 2, 4, 6, 8, 10, 12 and 14 min). Trials were carried out using a 150 mL enzyme solution (without substrate) treated with a constant ultrasound amplitude of 80%. At time intervals ranging from 0 to 14 minutes, samples were taken to test residual α-amylase activity.

A test to determine the effect of ultrasonic treatment versus enzyme assay was carried out to determine the effect of ultrasonic treatment on α-amylase activity at different enzyme concentrations. A concentration range for α-amylase was tested, from 25 to 500 mg/L, with values of 25, 50, 100, 150, 200, 250, 300, 350, 400, 450 and 500 mg/L. The impact of the incubation temperature prior to ultrasonic treatment was evaluated by heating a 150 mL enzyme solution to temperatures between 65 °C and 95 °C for 5 minutes.

The impact of various common chemical compounds on α-amylase activity was assessed by incorporating them into the reaction mixture. At a final concentration of 1 mM, the following substances were tested: KCl, AgNO_3_, MgSO_4_, CaCl_2_, MnCl_2_, FeSO_4_, CoCl_2_, CuSO_4_, ZnSO_4_, Pb(NO_3_)_2_, AlNH_4_(SO_4_)_2_, CdCl_2_, EDTA and NaCl; additionally, the effects of sodium dodecyl sulfate (SDS), ethylenediaminetetraacetic acid (EDTA), and β-mercaptoethanol (2-ME) were assessed by adding them at a final concentration of 10% [50], while enzymatic assays were carried out both in the absence and presence of these compounds under standard conditions, using phosphate buffer and ultrasonic treatment as previously described.

In addition, the effect of the calcium ion concentration was tested by adding CaCl_2_ to the reaction mixture at final concentrations ranging from 0.5 to 10 mM.

All experiments were performed with three replicates. The resulting data were subjected to analysis of variance (ANOVA) using GraphPad Prism 9 software (version 9.5.4, 2023). Fisher’s least significant difference (LSD) was used to determine statistically significant differences between means. A *p*-value of less than 0.05 was considered statistically significant.

### 3.3. Optimization of α-Amylase Activity with the RSM Method Using the Box–Behnken Design

Based on preliminary OFAT experiments, four process parameters were identified as potentially influential on ultrasound-mediated α-amylase modulation:

- Factor A—Ultrasonic Amplitude (%): Represents the intensity of ultrasonic energy delivered to the enzyme solution, expressed as a percentage of the sonicator’s maximum output. Higher amplitudes generate more intense cavitation phenomena.
- Factor B—Treatment Time (min): The duration of continuous ultrasonic exposure. Longer treatment times increase cumulative energy input but may also promote progressive enzyme denaturation.
- Factor C—Incubation Temperature (°C): The temperature at which the enzyme solution is maintained during ultrasonic treatment. Temperature affects both enzyme thermostability and the physical properties of cavitation bubbles.
- Factor D—Calcium Concentration (ppm): The concentration of Ca^2+^ ions in the reaction medium. Calcium is known to stabilize the α-amylase structure by binding to specific sites on the enzyme.

These four factors were selected because they represent distinct mechanisms of influence: mechanical stress (amplitude), cumulative exposure (time), thermal stress (temperature), and biochemical stabilization (calcium). Their combined effects were expected to determine whether ultrasound treatment would activate or inactivate the enzyme.

The response surface method (RSM), based on the Box–Behnken design (BBD), was used to optimize the levels of key variables in the experimental domain and to assess potential interactions between them. Based on this design, four independent variables were selected for the study (Table 4). The design consisted of 27 experimental runs including 24 factorial points and 3 center point replicates. Independent variables were coded at three levels: low (−1), center (0), and high (+1). The four factors included in the design are:

- Factor A—Amplitude: Ultrasonic amplitude ranging from 40% to 80%, representing the percentage of maximum sonicator output power.
- Factor B—Treatment Time: Duration of ultrasound exposure from 1 to 15 minutes.
- Factor C—Temperature: Enzyme incubation temperature prior to activity assay, ranging from 65 °C to 95 °C.
- Factor D—[Calcium]: Calcium ion concentration in the reaction medium, from 0 to 200 ppm (added as CaCl_2_).

These factors differ by their physical meaning and by their role in enzyme modulation. Amplitude and treatment time describe the ultrasonic treatment conditions, whereas incubation temperature and calcium concentration relate to the thermal environment and enzyme stability.

The experimental matrix of the Box–Behnken design (BBD), which evaluated the effects of four independent variables, ultrasound amplitude (A), treatment time (B), incubation temperature (C), and calcium concentration (D), on the residual α-amylase activity (%), is presented in Table 1. A total of 27 experimental runs were performed, including three central repeats, with each run performed in triplicate to ensure reliability and reproducibility (Table 1). This three-level, four-factor design (k = 4) allowed the investigation of both individual and interactive effects of the selected variables on the residual activity of thermostable α-amylase. The experimental matrix was generated using Design-Expert 10^®^ software (version 13.0.5.0, Stat-Ease Inc., Minneapolis, MN, USA), which facilitated both the construction of the design and subsequent regression analysis. The total number of experiments (*N*) required for the BBD was determined using the standard equation for such designs (Equation (3)).

(3) $$N=2k\cdot (k-1)+C_{0}$$

In which:

*k* denotes a number of independent variables (factors);

*C*_0_ is a number of central point replicates (for this study, *C*_0_ = 3).

During each experimental run, the α-amylase activity has been measured as a response variable. A second-order polynomial regression model was employed to evaluate the relationship between the input variables and the response. The model is expressed as follows (Equation (4)):

(4) $$R=\beta_{0}+\sum_{i=1}^{n}\beta_{i}X_{i}+\sum_{i=1}^{n}\beta_{ii}X_{i}^{2}+\sum_{i=1}^{n}\sum_{j=1}^{n}\beta_{ij}X_{i}X_{j}+\varepsilon$$

In this equation, *R* is the predicted response (residual α-amylase activity), *X_i_* are expressed in terms of the variables studied, where *i* varies from 1 to *k*. *β*_0_: constant term of the regression equation; *β_i_*: linear effects; *β_ij_*: interaction effects; *β_ii_*: quadratic effects.

## 4. Conclusions

The present study demonstrates that ultrasound treatment can modulate thermostable α-amylase activity in both activation and inactivation directions, depending on the specific combination of processing parameters.

The results obtained using the classical one-factor-at-a-time (OFAT) approach showed that moderate ultrasound amplitudes (60–80%) enhanced enzyme activity relative to the untreated control. In contrast, prolonged sonication and elevated temperatures led to progressive inactivation. These effects are consistent with the combined mechanical and thermal stresses associated with acoustic cavitation.

The influence of metal ions, particularly calcium, was found to be temperature-dependent. Under mild thermal conditions, calcium contributed to the preservation of enzyme activity. However, this stabilizing effect diminished at higher temperatures, where residual activity decreased substantially.

Response surface analysis using a Box–Behnken design confirmed that the incubation temperature and calcium concentration were the dominant factors influencing residual α-amylase activity. Their interaction significantly affected enzyme stability, whereas ultrasound amplitude and treatment time exhibited less pronounced independent effects within the tested experimental domain.

Model validation experiments confirmed that moderate ultrasonic conditions combined with appropriate calcium levels promoted enhanced enzyme activity, whereas high temperatures in the absence of calcium resulted in near-complete inactivation. These findings support the initial hypothesis that α-amylase activity responds in a parameter-dependent manner, with enhancement under controlled ultrasonic conditions and destabilization under more severe thermal–acoustic environments.

Overall, this study provides clear experimental evidence that ultrasound-assisted modulation of thermostable α-amylase is feasible and tunable. Such controlled regulation offers potential applications in food processing and industrial bioprocessing, where either enhanced catalytic performance or targeted enzyme inactivation may be required.

## Figures and Tables

**Table 1:** BBD matrix and observed residual α-amylase activity.

	Factor A	Factor B	Factor C	Factor D	Response
Run	Amplitude	Treatment Time	Temperature	[Calcium]	Residual activity
	%	Min	°C	ppm	%
1	60	15	80	0	11.86
2	40	8	95	100	25
3	40	8	80	200	54
4	60	8	95	200	50
5	60	1	80	0	12
6	60	8	80	100	16
7	80	15	80	100	31
8	60	8	80	100	25
9	60	15	80	200	44
10	60	8	80	100	31
11	80	8	65	100	51
12	80	8	80	0	12
13	60	1	80	200	59
14	80	8	80	200	56
15	60	15	95	100	36
16	80	1	80	100	28
17	60	8	95	0	3
18	60	1	95	100	23
19	60	8	65	200	76
20	40	1	80	100	27
21	60	8	65	0	15
22	60	15	65	100	49
23	60	1	65	100	69
24	80	8	95	100	32
25	40	8	65	100	52
26	40	15	80	100	29
27	40	8	80	0	13

**Table 2:** ANOVA for quadratic model fitted to the residual α-amylase activity (%).

Source	F-Value	*p*-Value
Model	15.15	<0.0001 significant
A-Amplitude	0.1935	0.6678
B-Time	0.5685	0.4654
C-Temperature	39.57	<0.0001
D-[Calcium]	143.33	<0.0001
AB	0.0058	0.9405
AC	0.3716	0.5535
AD	0.0523	0.8230
BC	6.32	0.0272
BD	1.28	0.2796
CD	1.14	0.3071
A^2^	1.52	0.2409
B^2^	2.64	0.1304
C^2^	18.99	0.0009
D^2^	1.11	0.3137
Residual		
Lack of Fit	0.7065	0.7124 not significant
R^2^	0.9465	
Adjusted R^2^	0.8840	
Predicted R^2^	0.7331	

**Table 3:** Validation of the quadratic model for studying the effect of ultrasound using the response surface method (RSM).

	Number	Amplitude	Incubation Time (min)	Incubation Temperature °C	[Calcium] mM	Residual Activities (%)
α-amylase activation Solutions	1	60	11	40	155	153
	2	40	5	30	110	229
	3	70	4	35	190	212
	4	40	14	25	20	188
	5	50	4	40	170	169
	6	50	10	25	74	205
α-amylase inactivation Solutions	1	50	3	90	00	1
	2	60	2	90	00	1

**Table 4:** Coded levels of independent variables in the Box–Behnken design.

Factor	Name	Units	Type	SubType	Coded Low	Coded High	Mean
A	Amplitude	%	Numeric	Continuous	−1 ↔ 40	+1 ↔ 80	60
B	Treatment Time	Min	Numeric	Continuous	−1 ↔ 1.00	+1 ↔ 15	8.00
C	Temperature	°C	Numeric	Continuous	−1 ↔75	+1 ↔ 95	85
D	[Calcium]	ppm	Numeric	Continuous	−1 ↔ 0.00	+1 ↔ 200	100

## Data Availability

The original contributions presented in this study are included in the article. Further inquiries can be directed to the corresponding authors.

## Abbreviations

The following abbreviations are used in this manuscript:

ANOVA	Analysis of variance
BBD	Box–Behnken design
DNS	3,5-dinitrosalicylic acid
OFAT	One factor at a time
RSM	Response surface methodology
